# Age-specific vulnerability and high prevalence of delirium in pediatric intensive care based on a prospective cohort study

**DOI:** 10.1038/s41598-024-82684-1

**Published:** 2024-12-28

**Authors:** AbdulRahman AlDaithan, Naila Shaheen, Eidah Alahmari, Abeer Al Smari, Arwa Al Ahmadi, Abdulaziz Almalahi, Msaed Alotaibi, Abdullah AlGhuraibi, Abdulaziz Alhusaini, Abdullah Bin Shaman, Tarek Hazwani

**Affiliations:** 1 King Abdullah International Medical Research Center, Riyadh, Saudi Arabia; 2 College of Medicine, King Saud bin Abdulaziz University for Health Sciences, Riyadh, Saudi Arabia; 3 Department of Pediatrics, King Abdulaziz Medical City, Ministry of National Guard-Health Affairs, P.O.Box 22490, Riyadh, 11426 Saudi Arabia; 4 Nursing Service, Ministry of National Guard-Health Affairs, Riyadh, Saudi Arabia; 5 College of Pharmacy, King Saud bin Abdulaziz University for Health Sciences, Riyadh, Saudi Arabia; 6 Pharmaceutical Care Service, Ministry of National Guard - Health Affairs, Riyadh, Saudi Arabia; 7 King Saud bin Abdulaziz University for Health Sciences, Riyadh, Saudi Arabia

**Keywords:** Delirium, Children, Pediatric, Critical care, Intensive care, Paediatrics, Paediatric research

## Abstract

Delirium, a neuropsychiatric syndrome characterized by acute disruptions in attention and awareness, significantly impacts children in Pediatric Intensive Care Units (PICUs), leading to prolonged hospitalization, increased infection risk, and dependence on mechanical ventilation. Despite growing recognition, its true burden and risk factors in children remain poorly understood. This prospective cohort study investigated the prevalence, characteristics, and potential therapeutic targets for delirium in 890 children admitted to a tertiary PICU between January and December 2022. Delirium was screened every 12 hours using the validated Cornell Assessment of Pediatric Delirium (CAPD). We analyzed data on demographics, comorbidities, medications, interventions, and clinical outcomes to identify associations with the development of delirium. Our study revealed a high prevalence of delirium, affecting 69.4% (95% CI: 66.33–72.3) of admitted children. Notably, infants were disproportionately affected, accounting for 33.5% of delirium cases. Respiratory diagnoses were significantly associated with delirium (78.6%), while oncology cases had the lowest prevalence (29.4%). Opioid use was identified as a risk factor, increasing the risk of delirium by 45.2%. Furthermore, 97.6% of children with withdrawal syndrome also experienced delirium, highlighting a strong association between these conditions. Delirium was significantly associated with longer PICU stays, and all 20 mortalities during the study period occurred in delirious patients. The adjusted odds ratios from multi-level regression modeling further elucidated the risk factors associated with the development of delirium. This study demonstrates a high prevalence of delirium in PICUs, with infants and those with respiratory diagnoses being particularly vulnerable. Opioid use and withdrawal syndrome emerged as risk factors. Further research is needed to elucidate the mechanisms underlying these associations and develop targeted interventions to prevent, manage, and improve outcomes for children suffering from delirium in critical care settings.

## Introduction

Delirium, a neuropsychiatric disorder characterized by acute disruptions in attention and awareness, significantly impacts critically ill children in Pediatric Intensive Care Units (PICUs). Estimates suggest that up to 65.6% of children admitted to PICUs experience delirium, highlighting its prevalence and potential consequences^1,2^. This complication of critical illness extends beyond immediate symptoms, contributing to poor outcomes such as prolonged ICU stays, increased infection risks, and dependence on mechanical ventilation^3–7^. While research in adult ICUs has established a clear link between delirium and increased mortality, its impact on pediatric patients remains under-investigated, with only a few studies exploring this association^8,26^. Days of delirium have been shown to increase mortality in the pediatric population admitted to the PICU according to univariate analysis^27^. Additionally, in adult ICUs, days of delirium were associated with higher one-year mortality after adjustment for relevant covariates^9^.

Despite its considerable impact, pediatric delirium has historically received less attention compared to its adult counterpart. Prior to 2009, research primarily consisted of isolated case reports, offering limited insights into the causes, incidence, and risk factors associated with pediatric delirium^10^. However, the availability of validated assessment tools since 2009, such as the Cornell Assessment of Pediatric Delirium (CAPD), the Preschool Confusion Assessment Method for the ICU (pCAM-ICU), and the Sophia Observational Withdrawal Symptom Scale–Pediatric Delirium (SOWS-P), has significantly improved our ability to accurately identify and measure pediatric delirium^3,11–14^. This advancement has yielded more precise estimates of its prevalence, duration, and presentation, with studies reporting a wide range from 15.9 to 65.6%^15–17^. Notably, mechanically ventilated children exhibit an even higher prevalence, ranging from 53 to 74%^2,18^.

Furthermore, certain characteristics within the pediatric population appear to increase susceptibility to delirium. Children under two years of age, those requiring mechanical ventilation, and those exposed to medications such as benzodiazepines, narcotics, vasopressors, anti-epileptics, or restraints are at significantly higher risk compared to others^4,5,7,18,19^. Moreover, respiratory system involvement on admission or the presence of comorbidities has been shown to be associated with a higher percentage of delirium compared to other system involvements^27^.

Despite the progress made in recent years, additional research remains crucial to further elucidating the prevalence of delirium in pediatric patients. Strengthening the evidence base concerning the risk factors associated with pediatric delirium is essential for optimizing prevention and management strategies.

Therefore, this study aims to estimate the prevalence of delirium and to identify the predisposing risk factors associated with an increased risk of delirium among children admitted to the PICU. This research will contribute to a deeper understanding of this prevalent and significant condition.

## Materials and methods

### Design, setting, participants, and variables

This prospective cohort study was conducted in the Pediatric Intensive Care Unit (PICU) at King Abdullah Specialist Children’s Hospital (KASCH) in Riyadh, Saudi Arabia. KASCH, a tertiary academic center in Riyadh, houses a 30-bed closed medical and surgical PICU, admitting approximately 1000 patients annually.

The study included patients aged 0–14 years, as 14 years is the upper limit for PICU admission at our institution. All patients admitted to the PICU between January 1, 2022, and December 31, 2022, were assessed for delirium using the Cornell Assessment of Pediatric Delirium (CAPD)^13^. Scoring was conducted from admission day (day 0) until the patient’s discharge, with nurses documenting scores every 12 h at the end of their shifts.

A CAPD score of 9 or above was considered positive. Our unit employs a sedation protocol for mechanically ventilated patients, offering two regimens: continuous infusion of morphine and midazolam, or infusion of fentanyl and midazolam. The treating physician selects the appropriate regimen for each patient. All sedated patients were monitored with Comfort-B score for the level of sedation, while the Withdrawal Assessment Tool (WAT) was used to assess withdrawal syndrome. The COMFORT-B scale was not applied during periods when patients received neuromuscular blockade infusions. Comfort-B score of 6–10, 11–22 and 23–30 indicate excessive sedation, adequate sedation and insufficient sedation, respectively. The WAT assesses withdrawal symptoms on a scale of 0–12, with 0 indicating no withdrawal and 3 or higher signifying withdrawal syndrome.

### Data sources

The Comfort-B score was utilized to assess patients’ sedation and comfort status by integrating delirium monitoring through the CAPD score into the Patient Health Information System (HIS). PICU nurses received comprehensive training from one of the six delirium champion nurses under the supervision of the chief nurse. Competency assessments ensured scoring standardization.

Patients were identified through the PICU registry, and their medical charts were reviewed for demographic data, pre-existing comorbidities, clinical information, and pharmaceutical details. Delirium prevalence in the PICU was assessed, considering patient age, gender, use of restraints, and exposure to various medications, either through infusion or intermittent dosing (benzodiazepines, opioids, vasopressors). Patient outcomes were categorized as discharge to the high dependency unit (HDU), ward, home, or mortality, with risk factors extracted from HIS patient records.

A total of 1,344 patients were screened for the study. Of these, 454 patients were excluded due to incomplete data, admission duration of less than 24 h, or admission solely for procedural sedation. For the remaining 890 patients, the CAPD score was retrieved from the electronic medical records. All other relevant variables were also extracted.

### Ethical statement

Ethics approval for the study was obtained from King Abdullah International Medical Research Center Institutional Review Board (IRB) prior to data collection. As no identifiers were collected, the IRB considered the study exempt from the need for informedconsent.

### Statistical analysis

The categorical variables were summarized as frequencies and percentages, and comparisons among groups were made using the chi-square test. Continuous variables were summarized as means and standard deviations. Since the continuous data were not normally distributed, continuous variables were compared using the Wilcoxon rank sum test. The prevalence of delirium was reported as a percentage along with the corresponding 95% Wilson confidence interval. For the multivariate analysis, logistic regression was employed to identify the risk factors for developing delirium in children admitted to the PICU. The outcome variable was the presence of delirium, and the model’s probability was based on this outcome. Independent factors were identified based on clinical judgment and significant factors from univariate analysis. Results were reported as adjusted odds ratios with corresponding 95% confidence intervals. A P-value of less than 0.05 was considered significant. All analyses were performed using SAS software, version 9.4.

## Results

### Cohort characterization

A total of 890 patients admitted to KASCH in the PICU from January 2022 to December 2022 were assessed for delirium. There was a statistically significant difference between gender and age across the groups (delirium vs. no delirium). The mean age of patients in the delirious group was 35.2 months, compared to 82.8 months in the non-delirious group. Notably, 46.6% of delirium cases occurred in children up to 12 years old, while they formed 75.7% of the non-delirious population. Cardiac comorbidities (80.9%) were the most common in the delirious group, in contrast to oncology comorbidities (45.2%) in the non-delirious group, followed by neurological comorbidities (78.8%). The distribution of patients between the delirious and non-delirious groups was comparable in those admitted to either medical or surgical PICU units (delirium in Medical PICU 74.9% vs. non-delirium in Medical PICU 68.4%; delirium in Surgical PICU 25.1% vs. non-delirium in Surgical PICU 31.2%). Respiratory cases, constituting 78.6% (p = < 0.0001) of admissions, showed a high delirium prevalence, while oncology cases had the lowest incidence at 29.4% (p = < 0.0003) (Table 1).

**Table 1:** Characteristics of children admitted to PICU with/without Delirium.

	Delirium *n* = 618 (69.43%)	No Delirium *n* = 272 (30.56%)	*p*-value
Gender n(%)			
Male	355(57.44)	133(48.9)	0.018*
Female	263(42.56)	139(51.1)	
Age in months (mean ± SD)	35.21 ± 47.67	82.8 ± 54.28	< 0.0001*
Age Categories n(%)			
Newborns	89(14.4)	2(0.74)	
Infants	207(33.5)	22(8.09)	< 0.0001*
Children	288(46.60)	206(75.74)	
Teens	34(5.5)	42(15.44)	
Comorbidities n(%)			
Respiratory	181(70.1)	77(29.84)	0.766
Cardiac	131(80.86)	31(19.14)	0.0005*
Renal	70(67.31)	34(32.69)	0.615
Hepatic	35(71.43)	14(28.57)	0.751
Neurological	190(78.84)	51(21.16)	0.0002*
Oncology	23(54.76)	19(45.24)	0.034*
None	452(67.97)	59(26.22)	0.102
PICU admission Unit n(%)			
Medical	463(74.92)	187(68.75)	0.056
Surgical	155(25.08)	85(31.25)	
Admission Diagnosis			
Respiratory	319(78.57)	87(21.43)	< 0.0001*
Cardiac	19(70.37)	8(29.63)	0.915
Renal	13(61.9)	8(38.1)	0.448
Hepatic	15(83.33)	3(16.67)	0.196
Neurology	97(75.78)	31(24.22)	0.092
Oncology	5(29.41)	12(70.59)	0.0003*

Categorical variables were compared using Chi-square test.

Continuous variables were compared using Wilcoxon rank sum Test.

*Significant p-values.

### Delirium prevalence

The prevalence of delirium in the study cohort was 69.4% (95% CI: 66.33–72.3). This prevalence was higher in males compared to females 72.7% (95% CI: 68.63–76.51) (*p* = 0.018) (Table 2).

**Table 2:** Prevalence of Delirium in children admitted to PICU.

	%	95% CI (lower limit–upper limit)	*p*-value
Overall Prevalence			
Delirium	69.4	66.33–72.3	< 0.0001
Delirium by Gender			
Males	72.75	68.63–76.51	0.018*
Females	65.4	60.65–69.91	
Delirium by Age Groups			
Newborns	97.8	92.3–99.4	< 0.0001*
Infants	90.3	85.8–93.5	
Children	58.3	53.9–62.5	
Teens	44.7	34-55.9	

*Fischer exact test.

Furthermore, the prevalence of delirium was notably higher in newborns [97.8 (95% CI: 92.3–99.4)] and infants [90.3 (95% CI: 85.8–93.5)].

### Outcome data

 Of the patients, 565 (63%) were discharged to the ward, while 265 (30%) were transferred to the High Dependency Unit (HDU). The mortality rate was 2.25% occurring only in the delirious group (Table 3).

**Figure 1:**
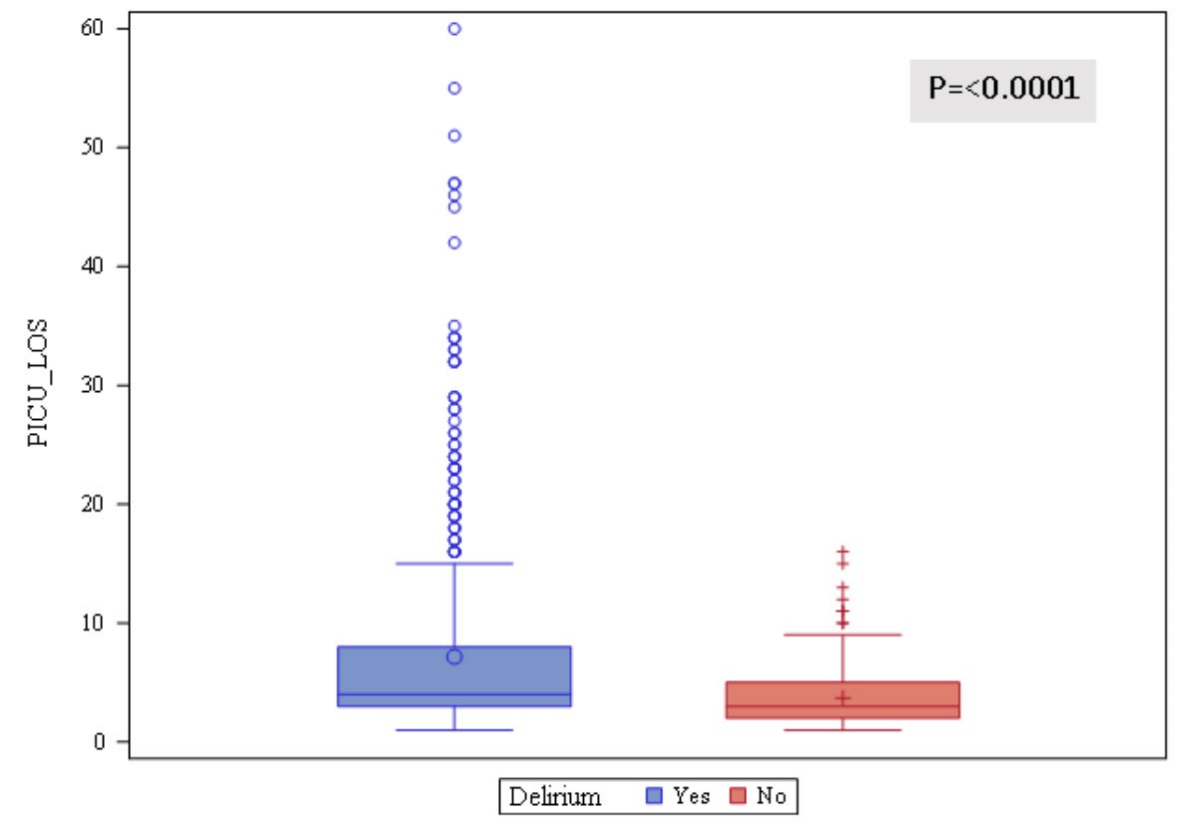
Delirium vs. Length of Stay (LOS).

The length of stay in PICU was longer among patients diagnosed with delirium compared to those who were not (Fig. 1).

Withdrawal syndrome was associated with delirium in 6.6% of the 618 delirium-positive patients, while vasopressors were used in 25.4% of the same group. Among restrained patients, 85.4% experienced delirium, while 14.6% did not (Table 3).

**Table 3:** Medication and Outcome Data by Delirium groups.

	Overall *n* = 890	Delirium *n* = 618	No Delirium *n* = 272	*p*-value
Benzodiazepines n(%)				
Yes	298(33.48)	258(41.75)	40(14.71)	< 0.0001
No	592(66.52)	360(58.25)	232(85.29)	
Opioids n(%)				
Yes	402(45.17)	297(48.06)	105(38.6)	0.009
No	488(54.83)	321(51.94)	167(61.4)	
Vasopressors n(%)				
Yes	188(21.12)	157(25.4)	31(11.4)	< 0.0001
No	702(78.88)	461(74.6)	241(88.6)	
On Antiepileptics n(%)				
Yes	130(14.61)	108(17.48)	22(8.09)	
No	760(85.39)	510(82.52)	250(91.9)	
Keppra n(%)				
Yes	114(12.81)	93(15.05)	21(7.72)	0.002
No	776(87.19)	525(84.95)	251(92.28)	
Phenytoin n(%)				
Yes	16(1.8)	14(2.27)	2(0.74)	0.169
No	874(98.20)	604(97.73)	270(99.26)	
Phenobarbital n(%)				
Yes	35(3.93)	33(5.34)	2(0.74)	0.001
No	855(96.07)	585(94.66)	270(99.26)	
Withdrawal n(%)				
Yes	42(4.72)	41(6.63)	1(0.37)	< 0.0001
No	848(95.28)	577(93.37)	271(99.63)	
Restrained n(%)				
Yes	48(5.39)	41(6.63)	7(2.57)	0.013
No	842(94.61)	577(93.37)	265(97.43)	
Outcome n(%)				
Discharged home	28(3.15)	13(2.1)	15(5.51)	< 0.0001
Discharged to HDU*	265(29.78)	206(33.33)	59(21.69)	
Discharged to ward	565(63.48)	371(60.03)	194(71.32)	
Died	20(2.25)	20(3.24)	0	
Others	12(1.35)	8(1.29)	4(1.47)	

*HDU (High dependency unit).

### Risk factors of developing delirium

The multivariate model exhibited good discriminatory power (C-statistic = 0.82). Among the analyzed risk factors, the use of benzodiazepines (AOR 0.295, p = < 0.0001), vasopressors (AOR 0.485, p = 0.003), and antiepileptic medication (AOR 0.445, *p* = 0.004) had a negative association with the development of delirium. In the age groups, newborns (AOR 84.98, p = < 0.0001) and infants (AOR 16.27, p = < 0.0001) had a very high likelihood of developing delirium compared to teens (Table 4).

**Table 4:** Risk factors of developing Delirium in Children admitted to the PICU.

	AOR*	95%CI (lower limit–upper limit)	*p*-value
Benzodiazepines (no vs. *yes*)	0.295	0.181–0.481	< 0.0001
Opioids (no vs. *yes*)	1.095	0.745–1.610	0.643
Withdrawal (no vs. *yes*)	0.119	0.016–0.910	0.040
Vasopressors (no vs. *yes*)	0.485	0.298–0.789	0.003
Antiepileptic Medication (no vs. *yes*)	0.445	0.254–0.778	0.004
Restrained (no vs. *yes*)	0.730	0.291–1.826	0.501
Age Groups (newborns vs. *teens*)	84.989	18.87-382.785	< 0.0001
Age Groups (infants vs. *teens*)	16.272	8.168–32.417	< 0.0001
Age Groups (children vs. *teens*)	1.774	1.025–3.069	0.041
Gender (females vs. *males*)	0.728	0.518–1.022	0.066

*AOR adjusted odd ratio.

Probability of the model is based on having the delirium.

Logistic regression model (c-statistics = 0.823).

## Discussion

The recognition of delirium in pediatric intensive care is gaining prominence, as shown by our single-center prospective cohort study, which reported a 69.4% prevalence of delirium. This rate is notably higher than 38% point prevalence reported by Traube et al.^19^. The elevated prevalence in our study may reflect the younger age distribution of our patient population, as younger age has been identified as a significant risk factor for delirium in critically ill children. Our findings align with existing research indicating that younger children, particularly newborns and infants, are more vulnerable to delirium due to developmental susceptibilities and the impact of critical illness on immature neurological systems. Additionally, our results reinforce age as an independent risk factor, underscoring the need for age-specific delirium prevention strategies in the PICU^19^. Benzodiazepine use was associated with a decreased likelihood of developing delirium in our PICU cohort, a finding that contrasts with much of the existing literature. In adult ICU populations, benzodiazepines are often considered deliriogenic, as their sedative effects are linked to increased delirium risk^20^. Studies by Smith et al. and Traube et al. suggest similar risks in pediatric populations, where the neurocognitive impacts of benzodiazepines may heighten delirium risk in developing brains^12,19^. However, recent research indicates that benzodiazepine effects may vary based on factors like dosage, duration, and patient-specific characteristics, suggesting that, in certain contexts, benzodiazepines might not strongly contribute to delirium^21,22^. Our findings may reflect the selective and controlled use of benzodiazepines in critically ill children, potentially impacting delirium risk differently. This nuanced association underscores the need for further research on how benzodiazepine dosing and patient characteristics might influence delirium risk in pediatric settings. While age is a critical factor, studies also associate opioid use with delirium, suggesting that each 1 mg/kg/day increase in opioid dosage raises delirium by 1.9 times^23^.

Our unit follows a sedation protocol for mechanically ventilated patients, employing two regimens that rely heavily on opioids. This protocol may significantly contribute to the high prevalence of delirium observed in our unit. Additionally, as a tertiary center, we manage complex referrals that often require extensive procedures and prolonged mechanical ventilation, which may further elevate delirium rates. Environmental factors, including sleep disturbances from uncontrolled alarm noise and disrupted sleep cycles, likely play a role, especially given the lack of a specific protocol to manage noise levels and promote sleep. Further investigation into these and other potential contributing factors is warranted.

Delirium is a common and serious complication in PICU patients, with contributing factors including age, pre-existing conditions, sedative use, and illness severity. The critical illness itself stands out as a major risk factor for delirium development^24,25^.

Our findings of a higher incidence of delirium in infants compared to older age groups align with previous research^26,27^. This observation suggests that rapid brain development in infants may increase their vulnerability to neuroinflammatory mediators released during peripheral infections, tissue destruction, or organ inflammation^28^.

Additionally, the notably higher prevalence of respiratory diagnoses in the delirious group in our study raises the possibility that hypoxic injury and oxidative stress from lung inflammation may contribute to delirium risk^28,29^.

Our study observed a correlation between the use of anti-epileptic medications (AEDs) and a lower incidence of delirium, though causation cannot be inferred from this analysis. Limited research specifically addresses the impact of AEDs on delirium in PICU patients; most studies focus on overall delirium management, with AEDs often considered as potential confounding factors rather than primary variables. While some AEDs possess sedative properties that might influence delirium risk, the relationship between AED use and delirium is complex and likely modulated by multiple factors^19,30^. Additionally, certain studies hint at an association between AEDs and delirium, underscoring the need for further investigation to clarify their role in this context.

Examining the impact of medications such as AEDs or vasopressors is complex, as a straightforward dichotomous approach may not fully capture their effects. Conducting a dose-response or sensitivity analysis might provide a clearer picture of these medications’ impact on delirium development.

Our findings are consistent with recent ESPNIC recommendations, showing that 97.6% of patients with withdrawal syndrome were diagnosed with delirium, which underscores the potential for delirium to be triggered in children experiencing withdrawal syndrome^31^.

Additionally, mortality in our study was exclusively associated with delirium; all 20 deceased patients had been diagnosed with delirium corroborating Traube et al.‘s findings of an independent link between delirium and mortality^18^.

However, several limitations of this study must be acknowledged. First, as a single-center study conducted over one year, the findings have limited generalizability. Second, high nurse turnover required ongoing education and knowledge reassessment, potentially impacting data consistency. Third, the data reviewed lacked key variables, including disease severity, mechanical ventilation parameters (which could represent important confounders like respiratory distress and sedation for device tolerance), ventilation duration, and pre-admission neurological function. These omissions may restrict our understanding of delirium risk factors and patient outcomes in pediatric populations. Additionally, reliance on electronic health records introduces concerns regarding data accuracy due to inconsistencies in documentation practices.

Future studies should explore delirium in specific age groups, particularly infants, and incorporate detailed data on disease severity, oxygen saturation index and sedative specifics, including opioid subtypes like morphine and fentanyl and their administration duration. Further research is warranted to examine these potential confounders and their influence on delirium prevalence and outcomes.

## Conclusions

In conclusion, this study underscores the high prevalence of delirium in PICUs, potentially influenced by factors such as opioids use, respiratory diagnoses, and younger age groups, particularly infants. Further research is warranted to clarify the role of antiepileptic medications and to deepen our understanding of the complex relationship among delirium, withdrawal syndrome, and mortality in this vulnerable patient population.

## Data Availability

The data underlying this article will be shared on reasonable request to the corresponding author.
